# Oral pigmented lesions: Clinicopathologic features and review of the literature

**DOI:** 10.4317/medoral.17679

**Published:** 2012-05-01

**Authors:** Rogério O. Gondak, Rogério da Silva-Jorge, Jacks Jorge, Márcio A. Lopes, Pablo A. Vargas

**Affiliations:** 1 DDS, MS, Oral Pathology Division, Department of Oral Diagnosis, Piracicaba Dental School, State University of Campinas, Piracicaba (Brazil); 2 DDS, PhD, Stomatology Clinic, Association of Dental Surgeons of Campinas (Brazil); 3 DDS, PhD, Oral Pathology Division, Department of Oral Diagnosis, Piracicaba Dental School, State University of Campina, Piracicaba (Brazil)

## Abstract

Diagnosis of pigmented lesions of the oral cavity and perioral tissues is challenging. Even though epidemiology may be of some help in orientating the clinician and even though some lesions may confidently be diagnosed on clinical grounds alone, the definitive diagnosis usually requires histopathologic evaluation. Oral pigmentation can be physiological or pathological, and exogenous or endogenous. Color, location, distribution, and duration as well as drugs use, family history, and change in pattern are important for the differential diagnosis. Dark or black pigmented lesions can be focal, multifocal or diffuse macules, including entities such as racial pigmentation, melanotic macule, melanocytic nevus, blue nevus, smoker’s melanosis, oral melanoacanthoma, pigmentation by foreign bodies or induced by drugs, Peutz-Jeghers syndrome, Addison´s disease and oral melanoma. The aim of this review is to present the main oral black lesions contributing to better approach of the patients.

** Key words:**Pigmentation, melanin, oral, diagnosis, management.

## Introduction

The color of oral pigmentation can vary depending on the quantity and depth or location of the pigment. Generally, the surface shows brown pigmentation and those located deeper are black or blue. Melanin is produced by melanocytes in the basal layer of the epithelium and is transferred to adjacent keratinocytes via membrane-bound organelles called melanosomes. Melanin is also synthesized by nevus cells, which are derived from the neural crest and are found in the skin and mucosa (1). The melanocytes are present in any region of the oral cavity and can be present in reactive, benign or malignant lesions.

In addition, pigmentation derived from foreign bodies, heavy metal poisoning or drugs may also promote pigmented lesions, which can vary in intensity and extension, and can occur in any sites of the oral cavity. The clinical history, symmetry and uniformity of the lesion are crucial in determining the clinical differential diagnosis.

-Physiological pigmentation

Physiological pigmentation is common and results from an increase in the production of melanin pigment by the melanocytes (2). Darker skinned individuals are more commonly affected. The color of physiological pigmentation can range from light brown to almost black. Phy-siological pigmentation increases with age, and color intensity can be influenced by smoking, hormones and systemic medications (3). The attached gingiva is the most common location, but physiological pigmentation can be noted anywhere in the oral cavity, including the tips of the fungiform papillae on the dorsal tongue and the diagnosis of physiological pigmentation normally is made clinically and do not need any treatment (4). However, when it is performed, demonstrate increased melanin pigmentation of the basal layer, as well as occasional incontinent melanin and/or melanophages in the superficial lamina propria without increasing number of melanocytes.

-Post inflammatory pigmentation

Long-standing inflammatory mucosal diseases, such as oral lichen planus, pemphigus or pemphigoid can cause mucosal pigmentation (3). The pathogenesis of post inflammatory pigmentation remains unclear (5) and can be seen more frequently in dark-skinned individuals. Clinically, multiple brown–black pigmented areas are noted adjacent to reticular, erosive or vesicular lesions. Microscopically, there is increased production of melanin by the melanocytes and accumulation of melanin-laden macrophages in the superficial connective tissue (1). Generally, the resolution of the inflammatory pro-cess allows the cessation of oral pigmentation.

-Melanotic macule

**Figure 1:**
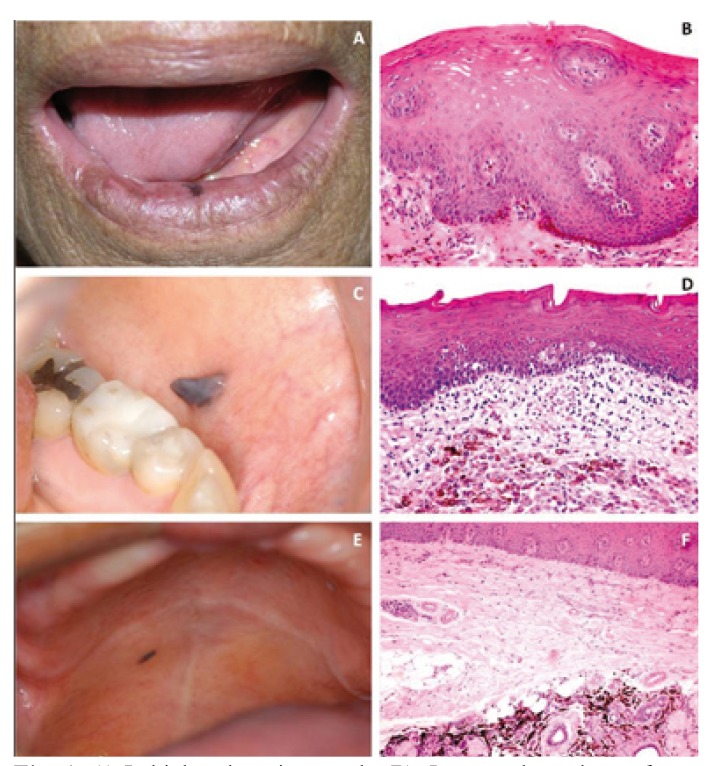
Labial melanotic macule. B). Increased numbers of me-lanocytes along the junctional zone (H&E, x 200). C) Intraoral me-lanocytic nevus located on the left buccal mucosa. D) Nevus cells located within the connective tissue (H&E, x 200). E) Blue nevus located on the right posterior hard palate. F) Blue nevus showing proliferation of dendritic melanocytes, elongated and spindle-shaped in connective tissue deep (H&E, x 100).

The oral melanotic macule is a small, well-circumscribed, brown-to-black that occurs commonly on the lips and gingiva, followed by the palate and buccal mucosa (Fig. 1). Patients age ranges from 4 to 98 years (mean 43.7) with predilection for females (1.9:1). Histologically, it is characterized by in situ increased production of melanin by basal melanocytes with normal morphological features (6) (Fig. 1). Melanin pigment is also observed in melanophages in the upper portion of the lamina propria (6). Generally a biopsy is recommended to distinguish melanotic macule from other oral melanocytic lesions.

-Melanocytic nevus

Melanocytic nevi are much less common on the oral mucosa than skin. Clinically, oral nevi are small, well circumscribed mac-ules but commonly appear as slightly raised papules (Fig. 1). They can be brown, bluish-gray, or almost black and occasionally non-pigmented (7). Regarding etiology and pathogenesis, most studies have focused on cutaneous lesions. It is now clear that melanocytic nevi constitute benign neoplasms of cutaneous melanocytes, which frequently harbour oncogenic serine/threonine-protein kinase B-Raf (BRAF) or, less commonly, neuroblastoma ras viral oncogene homolog (NRAS) mutations. Probably, oncogenic mutations drive the initial hyperproliferation that results in the formation of the nevi, while a subsequent growth-arrest response with the features of oncogene-induced cellular senescence accounts for the cessation of further growth (8).

Different from normal melanocytes, which are regularly interspersed as single cells among basal keratinocytes, forming the so called ‘‘epidermal-melanin unit’’, nevomelanocytes tend to cluster in compact so called theques (9). In view of the many histo-logic similarities, it seems plausible that the pathogenesis of oral melanocytic nevus be similar to that of the cutaneous lesions. Regarding morphogenesis, the melanocytic proliferation can be divided into three phases: proliferation of benign neoplastic melanocytes along the submucosalmucosal junction (junctional nevus); migration of these cells to the underlying mesenchymal tissue (compound nevus); and loss of the junctional component of the nevi, so that all remaining nevomelanocytes are located within the subepithelial connective tissue stroma (subepithelial nevus) (Fig. 1) (9).

There are no reports of malignant transformation of intraoral nevi even in patients who have had mutiple nevi or congenital nevi. Biopsy is strongly advisable for any new oral pigmentation because an early melanoma may be mistaken as melanocytic nevi (10).

-Blue nevus

A blue nevus is a benign, acquired melanocytic lesion that typically presents as an asymptomatic, slate-blue or blue-black smooth-surfaced macule or papule and usually measures less than 6 mm in diameter (Fig. 1) (11). The vast majority of blue nevi develops on the skin and has also been identified in a variety of mucosal sites (12). However, oral mucosa is rarely affected by blue nevus (13).

The intraoral variant of blue nevus is typically identified between the third and fifth decades of life, with an average patient age at diagnosis of 38 years. Females tend to be more commonly affected than males. Two thirds of all intraoral blue nevi are found on the hard palate, and the buccal mucosa is the second most common site of presentation (12).

Blue nevus is characterized by a variety of histologic subtypes, although most are classified as either “common” or “cellular” (14). The common blue nevus, which is the most frequent subtype seen in the oral cavity is characterized by an intramucosal proliferation of elongated, bipolar, spindle-shaped melanocytes that are often grouped in short fascicles parallel arranged to the overlying epithelium (Fig. 1) (14). In contrast, a cellular blue nevus is usually characterized by an intramucosal, nodular proliferation of dendritic spindle-shaped, pigmented melanocytes, in addition to tightly-packed aggregates of larger oval-to-round melanocytes with pale cytoplasm and little or no melanin (14). From a clinical standpoint, common blue nevi are almost always inno-cuous lesions and rarely recur. Furthermore, rare cases of malignant melanoma have been reported to arise in a cellular blue nevi (14).

-Smoker’s melanosis 

Smoker’s melanosis occurs in 25 to 31% of tobacco users and is characterized by discrete or coalescing multiple brown macules that usually involve the attached mandibular gingiva on the labial side, although pigmentation of the palate and buccal mucosa has also been associated with pipe smoking (15). Smoking-associated melanosis is due to increased melanin production by melanocytes and its deposition within the basal cell layer and lamina propria. The microscopic appearance of melanosis is essentially similar to that seen in physiologic pigmentation or a melanotic macule (16). A gradual return to normal pigmentation over several months to years has been reported following smoking cessation (17).

-Black Hairy tongue

Black hairy tongue is a painless, benign disorder caused by defective desquamation and reactive hypertrophy of the filiform papillae of the tongue (18). The hairy appearance is due to elongation of keratinized filiform papillae that may show various colors from yellow-brown to black depending on extrinsic factors, ie, tobacco, coffee, tea, food, and intrinsic factors, ie, chromogenic organisms in the normal flora (19). The exact pathogenesis is unclear. Precipitating factors include poor oral hygiene, use of the antipsychotic drug olanzapine, a broad spectrum of antibiotics such as erythromycin, and therapeutic head and neck radiation. A biopsy is usually not necessary for diagnosis due to the typical clinical presentation. Scraping or brushing the tongue and smoking cessation enhance the resolution of black hairy tongue. Trimming of the papillae may be required in extreme cases (20).

-Oral melanoacanthoma

**Figure 2:**
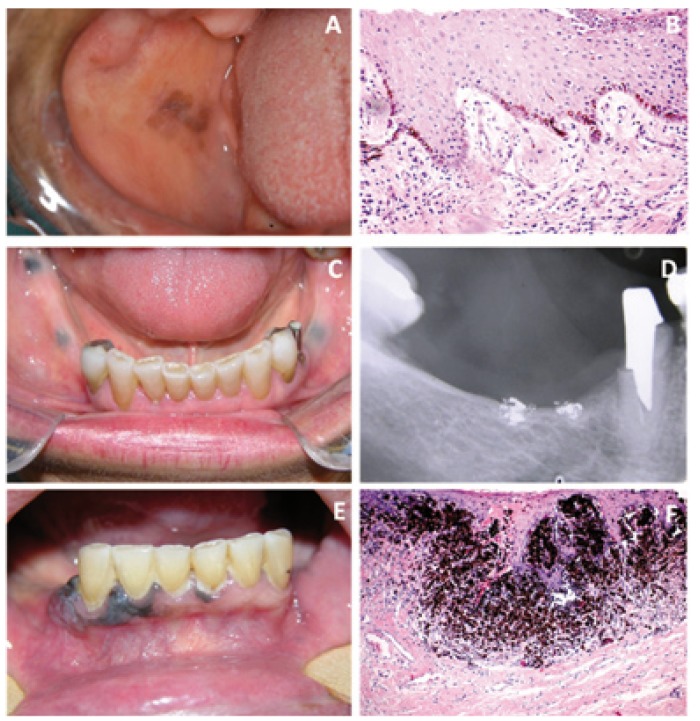
A) Melanoacanthoma on the right buccal mucosa. B) Me-lanoacanthoma showing proliferation of benign dendritic melanocytes scattered throughout the epithelium, acanthosis and spongiosis (H&E, x 100). C) Clinical aspect of amalgam tattoo on the gingival border bilaterally. D) Radiographic evaluation of amalgam tattoo. E) Oral melanoma located in gingival region. F) Histopathology of oral melanoma with atypical melanocytes, nuclear pleomorphism and hyperchromatism (H&E, x 100).

Oral melanoacanthoma is a rare, benign pigmented lesion, brown to brown-black, well circumscribed and similar to cutaneous melanoacanthoma, characterized by hyperplasia of spinous keratinocytes and dendritic melanocytes (Fig. 2). The pathogenesis of oral melanoacanthoma remains uncertain, although its clinical beha-vior is suggestive of a reactive origin. The most common intraoral sites are the buccal mucosa, lip, palate and gingiva. The average age of presentation is 28 years, mainly in blacks, with a strong female predilection (21).

A diagnosis of oral melanoacanthoma can be performed solely on the basis of the histological features and the lesion may regress after biopsy. Nevertheless, in order to emphasize the presence of melanin Fontana-Masson stain can be used. The immunohistochemical profile of oral melanoacanthoma is essentially limited to the melanocytic markers, but it is not necessary for the diagnosis (22). Immunohistochemical studies generally reveal diffuse nuclear and cytoplasmic immunoreactivity of the dendritic melanocytes with S-100 protein. These cells also exhibit moderate-to-diffuse cytoplasmic reactivity for HMB-45 (23). However, because of the heavy pigmentation of many of the dendritic cells, a reaction product may be difficult to visualize through the brown melanin.

-Foreign bodies 

Many metals have been implicated in the production of pigmentation in the oral region. These include lead, which produces characteristic generalized cutaneous ‘lead hue’ (described as a combination of pallor and lividity), and ‘lead lines’ on the gingiva (grey areas of discoloration below the gingival margins), mercury which can cause slategrey gingival hyperpigmentation, gold, bismuth and amalgam. Amalgam tattoo is caused by the presence of metallic material in the oral tissues. This most commonly follows the accidental implantation of dental filling material into the gingival or buccal mucosa (24). Amalgam tattoos are painless, gray-blue macules that range in size from a few millimeters to greater than 1 cm. The tattoo can be single or multiple. Most amalgam tattoos are located on the gingiva and edentulous mucosa (Fig. 2), but can also be seen on the hard palate, buccal mucosa, and floor of the mouth. Radiographic evaluation may be positive (Fig. 2). When mucosal pigment exhibits a blue-gray color in a patient that reports a history of dental amalgam restorations of either primary deciduous or permanent dentition, a biopsy may be unnecessary (1,2,24). However, if either of these criteria is not met, a biopsy is indicated. On histological examination, fine black granular or fibrillar material embedded in the connective tissue or in a perivascular location with little or no inflammatory response is seen. Foreign body giant cell reactions are uncommon (10).

-Drugs

In predisposed patients, drugs may cause an intraoral inflammatory reaction and subsequently induce postinflammatory hyper-pigmentation, a non-specific reaction that is the basis of pigmentary change seen in fixed drug reactions (25). Drugs such as arsenic can directly induce pigmentation by combining with sulphydryl groups in the epidermal cells causing promotion of the action of tyrosinase. Others such as the phenothiazines and minocycline may be deposited in the skin or mucosa and directly react with melanin to form a drug–pigment complex. Cotrimazole was the most common drug associated to oral pigmentation followed by tetracycline; however, many others have been implicated, including colchicines, ketoconazole, pyrimethamine and barbiturates (26). Fixed drug eruptions are more commonly seen in people with dark skin and often present as a slate brown color due to pigmentary incontinence of melanophages in the upper dermis. Pigmented macules of the tongue have also been described, occurring as the result of a fixed drug eruption (27).

Illicit drug use may also cause unusual areas of pigmentation. Westerhof et al. (28) reported striking dark pigmented macular areas on the dorsum of the tongue that they felt was the result of a fixed drug eruption following the inhalation of the heroin and methaqualone. Oral brownish pigmentation has also been reported in human immunodeficiency virus (HIV)-positive patients receiving treatment with zidovudine (AZT), ketoconazole and clofazamine (29).

-Peutz-Jeghers syndrome

**Figure 3:**
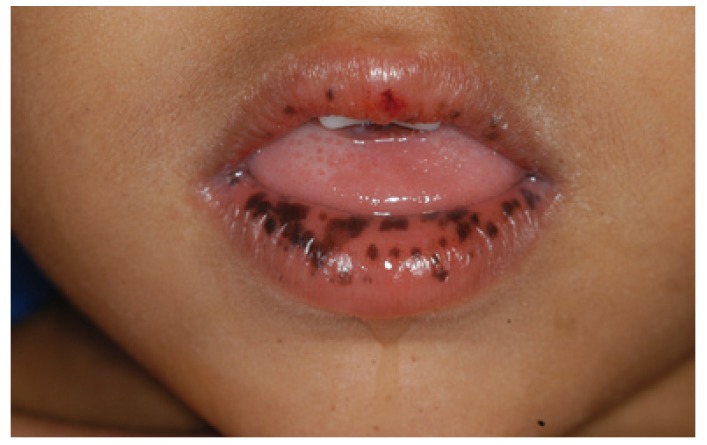
Peutz-Jeghers syndrome: black spots localized in the perioral area.

The Peutz-Jeghers syndrome (PJS) consists of mucocutaneous macules, intestinal hamartomatous polyposis, and increased risk of carcinomas of the gastrointestinal tract, pancreas, breast, and thyroid. PJS has autosomal dominant inheritance and germline mutations in the STK11/LKB1 gene on 19p13.3 are found in 30–70% of the cases, depending on the screening method, with considerable uncharacterized genetic heterogeneity remaining in this syndrome (30). Black-to-brown spots of less than 1 mm in size are typically localized on the lower lip and in the perioral area (Fig. 3). Intraoral, intranasal, conjunctival, and rectal pigmented lesions as well as spots localized on the acral surfaces may also be present (1,3). The oral lesions are benign and histologically characterized by an increase in melanin in the basal layer, without an obviously increased number of melanocytes (3). A fading or a disappearance of the spots is usually observed in older age (31).

-Addison´s disease and other endocrine disorders

Addison´s disease, is characterized by deficient production of hormones of the adrenal cortex, leading to increased production of adrenocorticotropic hormone (ACTH) (32). This may result in a diffuse dark pigmentation of the skin and the oral mucosa (1). Other signs and symptoms of Addison’s disease include anorexia, nausea, and postural hypotension. Lips, gingiva, buccal mucosa, hard palate, and tongue are usually involved. Pigmented lesions may be diffuse or localized and usually precede skin manifestations. Diffuse or discrete pigmentations of the lips and oral mucosa are sometimes observed in monostotic and polyostotic fibrous dysplasia (McCune-Albright syndrome), hyperthyroidism, and Nelson syndrome. Treatment of pigmented lesions of the oral mucosa associated with a systemic disorder is usually not required. The disappearance of oral lesions may follow the treatment of the underlying condition (1,32).

-Oral melanoma

Oral melanoma is rare, accounting for less than 1% of all oral malignancies. It is characterized by proliferation of malignant melanocytes along the junction between the epithelial and connective tissues, as well as within the connective tissue. The most common site is the hard palate, which accounts for about 40% of cases, followed by the gingiva (Fig. 2), which accounts for one third of cases. Other oral mucosal sites may also be affected. Oral melanoma is generally encountered between the fourth and seventh decades of life, with a greater incidence in men than in women. Clinically, oral melanoma may present as an asymptomatic, slow-growing brown or black patch with asymmetric and irregular borders or as a rapidly enlarging mass associated with ulceration, bleeding, pain and bone destruction. In approximately one-third of the cases, oral melanomas are characterized by a prolonged radial growth phase followed by a vertical growth phase; whereas others exhibit a faster progression into a vertical growth phase (33).

Histologically, the radial growth phase represents in situ and superficial melanoma and the vertical growth phase represent the nodular or invasive melanoma (34) (Fig. 2). The oral melanoma is not subdivided into the classical cutaneous melanoma categories, which include superficial spreading melanoma, nodular melanoma and acral lentiginous melanoma (34). The histologic microstaging system of Clark, used in cutaneous melanoma, cannot be applied to oral mucosa, because of the lack of histologic landmarks analogous to papillary and reticular dermis (34). The Tumor-Nodes-Metastasis clinical staging system (TNM) for oral melanoma recognizes three stages: stage I, primary tumor (T any N0 M0); stage II, metastatic tumor to regional lymph nodes (T any N1 M0); stage III, metastatic tumor to distant sites (T any N any M1) (35).

Treatment involves radical surgical excision with clear margins. This may be difficult to accomplish because of anatomic constraints and proximity to vital structures. Radiation and chemotherapy are ineffective, which adds to the difficulties associated with management of this malignancy. Distant metastases often affect the lungs, brain, liver, or bones. The prognosis for patients with oral melanoma is much worse than for those with cutaneous lesions, and the overall 5-year survival rate is 15% (36).

## Discussion

Diagnosis of pigmented lesions of the oral cavity and perioral tissues is challenging (1,37). Generally, the clinical aspects of pigmented lesions in the oral cavity are sufficient in establishing the diagnosis. However, in some cases, biopsy is necessary and occasionally, immunohistochemical stains such as melanocyte marker HMB-45 and macrophage marker CD68 may be required to prompt a correct diagnosis.

The ABCD checklist (asymmetry, border irregularities, color variegation, and diameter > 6 mm) that is commonly used to aid the identification of cutaneous melanoma may be of some utility in the clinical diagnosis of oral melanoma (38). Location on the palate increases the rate of suspicion of melanoma and usually requires a biopsy.

Solitary pigmented melanocytic lesions of the oral mucosa include oral melanotic macule, oral melanoacanthoma, melanocytic nevus, atypical melanocytic hyperplasia/proliferation, and melanoma (39). Melanotic macules are the most common lesions, affecting mainly the lip and gingiva, corresponding to 86.1% of solitary melanocytic lesions of the mouth (39). Oral mela-noacanthoma is usually a solitary lesion but multiple and bilateral lesions in the buccal mucosa have been reported (22). The oral lesions generally regress after removal of traumatic irritants or after excisional biopsy.

Oral melanocytic pigmentations have been reported in patients with Laugier-Hunziker syndrome (idiopathic lenticular muco-cutaneous pigmentations) and with Carney complex (spotty skin pigmentations, myxomas and endocrine overactivity). Human immunodeficiency virus infection has been associated with multiple usually well circumscribed melanotic macules locali-zed on the buccal and palatal mucosa, gingiva, and lips (40). However, the association may be only coincidental. The histopatho-logic appearance is similar to classical melanotic macules. It remains unclear whether such pigmentations are caused by the virus, therapy, or other factors (3).

Chronic inflammatory conditions, such as oral lichen planus, pemphigus, pemphigoid, and chronic periodontal disease, are sometimes associated with deposition of melanin within the connective tissue, resulting in a darkening of the mucosal area (3,37). The presence of lesions resembling melanotic macules of the palate in patients with lung diseases, including cancer, also has been reported, but there is lack of evidence of a true association. Melanin production and stimulation may also sometimes follow surgical procedures (37).

Many pigmented lesions can be clinically diagnosed based on size, shape, or color, along with the clinical information. Developing a differential diagnosis is imperative for a clinician faced with these lesions in order to appropriately treat the patient. Therefore, the establishment of effective clinical maneuvers in front of pigmented lesions of oral mucosa is crucial in the exclusion of possible malignancies.
